# Feasibility study for a randomized clinical trial of bupivacaine, lidocaine with adrenaline, or placebo wound infiltration to reduce postoperative pain after laparoscopic cholecystectomy

**DOI:** 10.1002/bjs5.50159

**Published:** 2019-03-26

**Authors:** A. T. Adenekan, A. A. Aderounmu, F. O. Wuraola, A. M. Owojuyigbe, A. O. Adetoye, D. Nepogodiev, L. Magill, A. Bhangu, A. O. Adisa

**Affiliations:** ^1^ Department of Anaesthesia and Intensive Care Obafemi Awolowo University Ile‐Ife Nigeria; ^2^ Department of Surgery Obafemi Awolowo University Ile‐Ife Nigeria; ^3^ Department of Anaesthesia and Intensive Care Obafemi Awolowo University Teaching Hospitals Complex Ile‐Ife Nigeria; ^4^ Department of Surgery Obafemi Awolowo University Teaching Hospitals Complex Ile‐Ife Nigeria; ^5^ National Institute for Health Research Global Health Research Unit on Global Surgery University of Birmingham Birmingham UK

## Abstract

**Background:**

Short‐term pain relief can be achieved by local anaesthetic infiltration of port sites at the end of laparoscopic surgery. This study aimed to assess feasibility of performing an RCT to evaluate short‐term postoperative analgesia after laparoscopic surgery in Nigeria using two local anaesthetics for port‐site infiltration *versus* saline placebo.

**Methods:**

This was a placebo‐controlled, patient‐ and outcome assessor‐blinded, external feasibility RCT. Patients undergoing elective laparoscopic cholecystectomy for symptomatic ultrasound‐proven gallstones were randomized into three groups: lidocaine with adrenaline (epinephrine), bupivacaine or saline control. The feasibility of recruitment, compliance with randomized treatment allocation, and completion of pain and nausea outcome measures were evaluated. Pain was assessed at 2, 6, 12 and 24 h after surgery using a 0–10‐point numerical rating scale (NRS) and a four‐point verbal rating scale. Nausea was assessed using NRS at the same time points. Clinical outcomes were assessed only in patients who received the correct randomized treatment allocation.

**Results:**

Of 79 patients screened for eligibility, 69 were consented and randomized (23 per group). Overall, compliance with randomized treatment allocation was achieved in 64 patients (93 per cent). All pain and nausea assessments were completed in these 64 patients. On the NRS, most patients had moderate to severe pain at 2 h (39 of 64, 61 per cent), which gradually reduced. Only six patients (9 per cent) had moderate to severe pain at 24 h.

**Conclusion:**

Recruitment, compliance with the randomized allocation, and completion of pain outcome measures were satisfactory. This study demonstrates the feasibility of conducting a surgical RCT in a resource‐limited setting. Registration number: ISRCTN 17667918 (https://www.isrctn.com).

## Introduction

Around 83 per cent of the world's population live in low‐ and low–middle‐income countries (LMICs)^1^. More than half of all surgical procedures completed worldwide take place in LMICs^2^. Conversely, most research is conducted in high‐income country settings^3^. Differences in clinical demands and resource constraints mean that research findings from high‐income settings cannot be translated directly to LMICs. Although an international priority‐setting exercise^4^ identified perioperative care as a key surgical research priority in LMICs, there are few ongoing perioperative trials in these environments. In a recent Cochrane review^5^ of wound infiltration of local anaesthetic in patients undergoing laparoscopic cholecystectomy, only one^6^ of the 26 studies identified was conducted in either a low‐ or lower middle‐income country. The review's finding that this is a safe and effective analgesic intervention may not be generalizable to low‐ or lower middle‐income countries.

Nigeria is a LMIC in West Africa where laparoscopic cholecystectomy has become established in the treatment of symptomatic gallstones^7^. Trials in high‐income settings^8^, ^9^, ^10^ have demonstrated that, compared with open surgery, a laparoscopic approach for cholecystectomy results in a shorter duration of hospital stay, better short‐term quality of life and quicker return to normal activities. Ambulatory surgery would be especially advantageous in Nigeria. Lacking a social safety net, many patients undergoing laparoscopic cholecystectomy have to stay overnight afterwards^7^, ^11^.

Pain is the most frequent perioperative symptom, with approximately one‐third of patients reporting pain intensity greater than 50 on a 100‐point visual analogue scale following laparoscopic cholecystectomy^12^. As postoperative pain can be a barrier to early discharge, to realize the full potential of laparoscopic cholecystectomy, analgesic strategies are required that ensure adequate postoperative pain control with minimal side‐effects, particularly in the first 24 h when pain is greatest. The use of non‐steroidal anti‐inflammatory drugs (NSAIDs) and local anaesthetic techniques provide superior analgesia with minimal side‐effects and spare the need for opioid administration^13^, ^14^.

There is no standardized practice across Nigeria for wound infiltration with local anaesthetic. Given the evidence from high‐income settings that local anaesthetic wound infiltration is safe and effective, there is equipoise to undertake a clinical trial in Nigeria. This randomized external feasibility study aimed to determine the possibility of delivering, in a resource‐limited setting, a full phase III trial of two commonly used local anaesthetics, lidocaine with adrenaline (epinephrine) and bupivacaine, infiltrated at laparoscopic port sites *versus* placebo, for early postoperative pain control after laparoscopic cholecystectomy.

## Methods

This was a single‐centre, placebo‐controlled, patient‐ and outcome assessor‐blinded, randomized, controlled, external feasibility study. The study protocol was approved by the Ethics and Research Committee of the Obafemi Awolowo University Teaching Hospitals Complex, Ile‐Ife, Nigeria (reference number IRB/IEC/0004553). As the study drugs are fully licensed in Nigeria, further study approval from the medicines competent authority was not required. This study is reported in accordance with the guidance set out in the CONSORT guidelines^15^. The trial was registered retrospectively (ISRCTN 17667918).

### Feasibility study objectives and outcome measures

This feasibility study aimed to evaluate: recruitment, to see whether informed consent could be taken and recruitment achieved to target; compliance, to see whether interventions could be delivered to patients as per randomized allocation; and outcome measurement, to see whether data on pain‐ and nausea‐related outcome measures could be collected at prespecified time points.

Success was evaluated by means of: recruitment, as the ability to consent and recruit 69 patients (23 per group) over a 24‐month period; compliance, as achieving the randomized treatment allocation for more than 90 per cent of randomized patients; and outcome assessment, as completion of pain and nausea assessments using numerical and verbal rating scales at 2, 6, 12 and 24 h after surgery, by at least 60 patients.

### Patient eligibility

Adult patients (aged 18 years or more) undergoing elective laparoscopic cholecystectomy for symptomatic gallstone disease were recruited. Patients were diagnosed as having symptomatic gallstone disease on the basis of a suitable clinical history and gallstones on ultrasound imaging. Only patients with ASA physical status class I or II were eligible. The following exclusion criteria were applied: pregnant women; acute cholecystitis confirmed on ultrasonography (gallbladder wall thickened by more than 5 mm and/or presence of pericholecystic fluid collection); history of allergy to bupivacaine, lidocaine or related local anaesthetic drugs; known peptic ulcer disease; known opiate addiction; or known contraindications to use of NSAIDs (such as bronchial asthma). When an abdominal drain was inserted or the operation was converted to an open procedure, the patient did not receive the allocated intervention and was withdrawn from follow‐up, so pain and nausea scores were not collected.

### Consent

Potentially eligible patients scheduled to undergo laparoscopic cholecystectomy were provided with a written patient information sheet and given the opportunity to ask questions. These information sheets were translated and available in English and Yoruba, the predominant languages in south‐west Nigeria. Once eligibility had been confirmed by the senior surgeon (the night before surgery, when patients are normally admitted for anaesthetic review), the patient was asked for written informed consent to participate in the study. Consent was obtained by an attending surgeon or surgical trainee. All investigators were trained by the chief investigator to take informed consent.

### Baseline techniques

#### *Surgery*

Elective laparoscopic cholecystectomy was performed under general anaesthesia by a single attending surgeon using two 10‐mm and two 5‐mm ports. This included hook diathermy to identify Calot's triangle and complete dissection of the liver bed. All procedures were carried out with locally adapted equipment, used to perform more than 400 laparoscopic procedures before the start of the study^16^.

#### *Anaesthesia*

Patients received premedication with intravenous Minijet® Atropine (UCB Pharma, Slough, UK), 0·01 mg/kg. Intravenous cefuroxime (Zinacef®; GlaxoSmithKline, Uxbridge, UK) 1·5 g, was administered as a prophylactic antibiotic. Anaesthesia was induced with fentanyl (Sublimaze® Injection; Janssen‐Cilag, Sydney, New South Wales, Australia) 1–2 µg/kg, and propofol (Nirfol® 1 per cent; Jawa House Compound, Lagos, Nigeria) 1–2 mg/kg. Tracheal intubation was facilitated with suxamethonium (Anectine®; GlaxoSmithKline) 1–1·5 mg/kg, and anaesthesia was maintained with isoflurane (Abbott Laboratories, Maidenhead, UK) 0·6–1·5 per cent in an oxygen–air mixture. Muscle relaxation was achieved with pancuronium (Pavulon®; Organon, West Orange, New Jersey, USA) 0·1 mg/kg, then topped up as appropriate. Gastric decompression was performed with a nasogastric tube that was inserted after intubation and removed at the end of surgery, before extubation. Residual neuromuscular blockade was reversed with Minijet® Atropine 1·2 mg and neostigmine methyl sulphate injection BP (Hameln Pharmaceuticals, Gloucester, UK) 2·5 mg in 1 ml. All patients received maintenance fluid during the operation.

#### *Analgesia*

Fentanyl boluses of 1–2 µg were given for intraoperative analgesia up to a maximum dose of 200 µg. All patients were given intramuscular diclofenac (Voltarol®; Novartis Pharmceuticals, Frimley, UK) 75 mg at the start of surgery, with further doses 12 and 24 h after surgery. Tramadol (Tramal® Injection; Seqirus, Auckland, New Zealand) 1·0 mg/kg intravenously or intramuscularly was used as an on‐demand postoperative rescue analgesic. Introduction of this agreed standardized postoperative analgesia protocol ensured that the anaesthetists who performed randomization did not prescribe patients' postoperative analgesia, and the surgical team was able to prescribe this safely while blinded to the patients' treatment allocations.

### Interventions

At the end of the procedure, patients had their skin, subcutaneous tissue, fascia and parietal peritoneum infiltrated with: lidocaine (3 ml of 2 per cent Xylocaine® with adrenaline; AstraZeneca, Luton, UK) in each 10‐mm port site and 2 ml in each 5‐mm port site (total 200 mg lidocaine with 1 : 1000 adrenaline); bupivacaine (3 ml of 0·5 per cent Marcain®; AstraZeneca) in each 10‐mm port site and 2 ml in each 5‐mm trocar site (total 50 mg plain bupivacaine); or 3 ml 0·9 per cent saline in each 10‐mm port site and 2 ml in each 5‐mm port site (control).

### Randomization and blinding

Patients were randomized in a 1 : 1 : 1 ratio between the three groups. Twenty‐three labelled cardboard cards were sealed in opaque envelopes for each patient in group 1 (lidocaine), group 2 (bupivacaine), and group 3 (control). These envelopes were shuffled and packed by an independent researcher not involved in the study. Envelopes were kept in a secure drawer in the operating room. Before the operation commenced (but after eligibility had been confirmed and informed consent taken), the anaesthetist opened the next envelope in sequence. Once the operating surgeon was about to extract the gallbladder laparoscopically, the anaesthetist prepared the solution based on the randomized allocation. This was recorded on a case report form and the solution was passed to the operating surgeon before wound closure. The operating surgeon, the assessor on the ward, and the patient were all blinded to the randomized allocation. This was not documented in any of the regular intraoperative or postoperative hospital records. The case report form was stored in a locked office within the operating department that was accessible by consultant anaesthetists if required (for instance in case of allergic reaction).

### Pain‐related outcome measures

Postoperative pain was assessed at 2, 6, 12 and 24 h after surgery. Two pain‐related outcome measures were recorded. Patients were asked to rate their pain on a numerical rating scale (NRS) from 0 (no pain) to 10 (worst pain), and on a four‐step verbal rating scale (VRS) of none, mild, moderate and severe pain. Nausea was assessed alongside the pain outcomes, using the NRS. These outcome measures have been validated previously for the assessment of pain in Nigeria^17^, ^18^. Assessments were performed by surgical residents who had attended training sessions led by a senior anaesthetist. Outcome assessors were blinded to patients' randomized treatment allocations.

### Safety and adverse events

This feasibility study recorded intraoperative complications, 24‐h postoperative complications, drug‐related allergic reactions and other drug‐related adverse events. All complications within 24 h were captured, including expected and unexpected adverse events.

### Sample size

As the purpose of the study was to assess whether recruitment to, and delivery of, a full trial would be feasible, no formal sample size calculations were performed. To assess feasibility of outcome assessment, the study aimed to collect outcome measures for 20 patients per trial arm (60 in total). To take into account intraoperative exclusions (such as conversion to open surgery) or losses to follow‐up, 23 patients were planned per arm (dropout rate 9 of 69 patients). Approximately three laparoscopic cholecystectomies are performed each month in the hospital, so this feasibility study aimed to recruit a total of 69 patients over 24 months.

### Data management and statistical analysis

Data were entered into a password‐protected database, held on a secure computer in a locked office by one of the authors. Numbers of patients recruited and allocations are reported as intention to treat (with a denominator of 69). As the intervention was not applied in patients converted to an open procedure or left with an abdominal drain, pain scores are reported only per‐protocol, from those receiving the correct allocation.

Outcomes relating to trial feasibility were described using basic statistics and tables. Data are reported as median (i.q.r.) values. As the main aim of the study was to assess trial feasibility, direct statistical comparison of pain scores was not planned. Mean point scores for NRS and VRS are reported, along with their 95 per cent confidence intervals. Statistical analysis was performed in SPSS® version 22 (IBM, Armonk, New York, USA).

## Results

**Figure 1:**
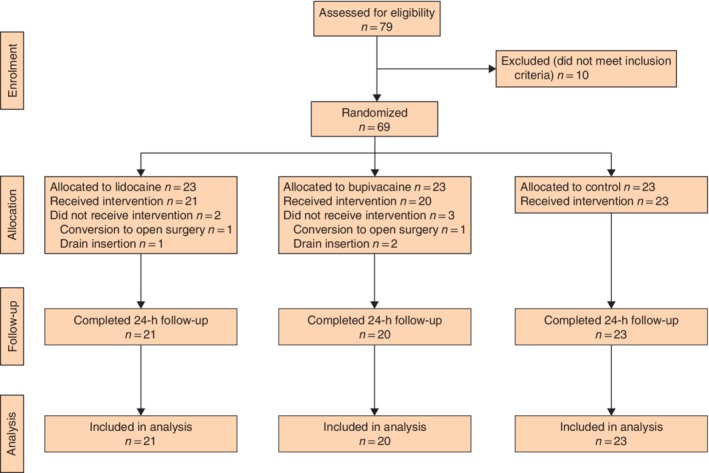
Flow diagram of patients from evaluation to completion of 24‐h follow‐up

Of 79 patients evaluated for inclusion, seven were excluded owing to previous NSAID‐induced gastritis or peptic ulceration, two because of sickle cell anaemia with opioid addiction, and one due to bronchial asthma. The CONSORT diagram illustrates patient flow through the study, including potentially eligible and excluded patients (*Fig*. 1).

### Compliance

Compliance with randomized treatment allocation was 21 of 23 in the lidocaine group (91 per cent), 20 of 23 in the bupivacaine group (87 per cent) and 23 of 23 in the control group (100 per cent). One patient each in the lidocaine and bupivacaine group was withdrawn before receiving the randomized treatment allocation due to conversion to open surgery. A further patient in the lidocaine group and two in the bupivacaine group were withdrawn from follow‐up because of abdominal drain insertion.

### Patient demographics

Overall, 59 of the 64 remaining patients (92 per cent) were women, and the median age was 44 (i.q.r. 36–57, range 20–85) years. Median BMI was 26·6 (i.q.r. 22·9–30·9) kg/m^2^. The median duration of surgery was 85 (i.q.r. 68–105) min. Baseline data were comparable across the three groups (*Table* 1).

**Table 1:** Baseline demographics by treatment allocation

	Lidocaine (*n* = 21)	Bupivacaine (*n* = 20)	Placebo (*n* = 23)
Age (years)*	43 (36–50)	47 (33–58)	43 (38–58)
Sex			
F	18 (86)	19 (95)	22 (96)
M	3 (14)	1 (5)	1 (4)
BMI (kg/m^2^)*	26·6 (20·9–28·5)	25·5 (22·7–39·2)	28·3 (23·5–32·4)
Duration of surgery (min)*	75 (70–100)	83 (65–95)	95 (70–120)

Values in parentheses are percentages unless indicated otherwise;

* values are median (i.q.r.)

### Clinical outcome measurement

**Figure 2:**
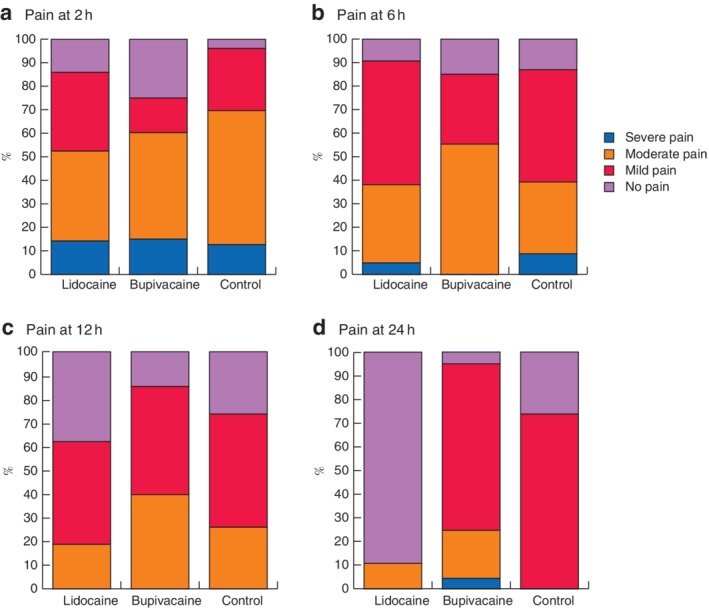
Pain scores on the verbal rating scale. Proportion of patients with severe, moderate, mild or no pain at **a** 2 h, **b** 6 h, **c** 12 h and **d** 24 h

NRS and VRS pain outcome measurements were complete for all patients, with outcomes recorded at all four prespecified time points. The overall mean pain score on the NRS at 2 h was 4·6 (95 per cent c.i. 3·9 to 5·2), with mean pain scores in the three groups ranging from 4·1 to 5·2 (*Table* 2). Pain scores reduced gradually over time, with the overall mean NRS score at 24 h being 2·0 (1·6 to 2·4). On VRS measurements, most patients had moderate to severe pain at 2 h (61 per cent, 39 of 64), which gradually reduced, with fewer than one in ten patients (9 per cent, 6 of 64) having moderate to severe pain at 24 h (*Fig*. 2).

**Table 2:** Pain scores on the numerical rating scale during the first 24 h after surgery

Time after surgery (h)	Lidocaine	Bupivacaine	Placebo
2	4·4 (3·3, 5·5)	4·1 (2·9, 5·2)	5·2 (4·1, 6·4)
6	3·4 (2·5, 4·3)	3·8 (2·8, 4·8)	3·8 (2·8, 4·8)
12	2·3 (1·6, 3·1)	3·1 (2·4, 3·8)	3·3 (2·4, 4·3)
24	1·4 (0·8, 2·0)	2·6 (1·6, 3·5)	2·0 (1·4, 2·6)

Values are mean (95 per cent c.i.).

All patients completed nausea scores. Few patients experienced significant nausea as captured by NRS nausea scores; at 2 h mean nausea scores ranged from 0·7 to 1·5 (*Table* 3), with only 13 of the 64 patients (20 per cent) rating their nausea level as higher than 1 of 10. By 24 h, 59 patients (92 per cent) reported no nausea, with the remaining five patients rating their nausea score as 1.

**Table 3:** Nausea scores on the numerical rating scale in the first 24 h after surgery

Time after surgery (h)	Lidocaine	Bupivacaine	Placebo
2	1·5 (0·2, 2·7)	0·7 (0·0, 1·3)	0·9 (0·1, 1·6)
6	1·5 (0·4, 2·5)	0·8 (0·0, 1·6)	0·4 (0·0, 0·8)
12	0·6 (0·1, 1·1)	0·5 (0·0, 1·2)	0·1 (0·0, 0·3)
24	0·1 (0·0, 0·2)	0·1 (0·0, 0·2)	0·0 (0·0, 0·1)

Values are mean (95 per cent c.i.).

### Safety and adverse events

One patient had intraoperative bile spillage, managed with copious irrigation. No other intraoperative or postoperative complications occurred by 24 h. There were no allergic reactions or drug‐related adverse events.

## Discussion

This randomized external feasibility study has shown that this surgical RCT is feasible in a resource‐limited setting. The study was completed on schedule. Compliance with randomized treatment allocation was high. Completion of pain scales at prespecified time points within the first 24 h after surgery demonstrated that measurement of patient‐reported outcome measures is also feasible. These objectives were achieved in spite of numerous resource limitations. It took 24 months to recruit 69 patients undergoing laparoscopic cholecystectomy, reflecting the low volume of these procedures in this setting. Additional challenges included interruptions in hospital services owing to industrial action and cancellation of procedures due to lack of surgical supplies. Lacking dedicated research staff, the surgical and anaesthetic teams oversaw all trial processes, including patient eligibility screening, consent, randomization and outcome assessment. The high recruitment rate was made possible by actively engaging surgical residents in the study, ensuring that there was always an investigator available to consent and randomize the patient. Although the recruitment rate may reflect social and cultural factors, it is likely that the in‐theatre intervention and short follow‐up period presented a low burden for participants.

There is no social‐security safety net in Nigeria to compensate patients for time off work. As patients are often the main financial providers for their family, a key priority is to return to work at the earliest opportunity. Compared with open surgery, minimal‐access surgery has been demonstrated to result in shorter hospital stays and earlier return to normal activities in LMICs^16^. Day‐case laparoscopic cholecystectomy would potentially help patients return to economic and caring activities sooner, while reducing the cost of the operation and thereby increasing access to surgery. This study has identified that postoperative pain is a barrier to the introduction of ambulatory laparoscopic cholecystectomy, as almost all patients experience moderate to severe pain in the first hours after the operation. This is in keeping with a previous study^12^ that identified that more than one‐quarter of patients rated their pain higher than 50 of 100 at 5 h after surgery.

This study has a number of limitations reflecting the resources available to support its design and conduct. Randomization was conducted using sealed envelopes, which is less robust than remote randomization, such as by telephone^19^. As patients can be difficult to contact after discharge, follow‐up was limited to 24 h while they remained in hospital. Randomization just before wound closure would have prevented protocol violations with patients being withdrawn from follow‐up due to conversion to open surgery or insertion of a drain. When this did occur, data ideally would have been collected for an intention‐to‐treat analysis. Finally, trial registration should have been completed before patient recruitment started. As the investigators develop research experience and collaborations, design and conduct of future randomized trials will improve and overcome these challenges.

A future phase III RCT could collect additional data for secondary endpoints. These could include pain over the first postoperative week. As most patients are discharged within the first 48 h, mobile phone technology may have a role in facilitating postdischarge follow‐up^20^. A full RCT would also collect cost and EQ‐5D™ (EuroQol Group, Rotterdam, the Netherlands) data in order to undertake a formal health economic analysis to establish the cost‐effectiveness of the intervention in a resource‐limited environment.

Evidence‐based analgesic strategies are needed to reduce very early postoperative pain, as at 12–24 h most patients experienced no pain, or only mild pain. A randomized placebo‐controlled trial^21^ of intraperitoneal and port‐site instillation found bupivacaine provided pain relief in the first 2 h after surgery, whereas another study^22^ found bupivacaine to be effective for up to 6 h after surgery. Intraperitoneal and port‐site levoropivacaine has also been found^23^ to reduce immediate postoperative pain and opioid use following laparoscopic cholecystectomy, as well as reducing length of hospital stay. Baseline data from the present study might be used to calculate a sample size for a large phase III trial to investigate strategies to reduce postoperative pain. Given the relatively low volume of laparoscopic surgery at this centre, collaboration with centres in other LMICs will be needed to deliver an adequately powered phase III trial. This feasibility study has developed this unit's capacity to contribute to leading a larger RCT in the future.
